# Effect of milk fat-based infant formulae on stool fatty acid soaps and calcium excretion in healthy term infants: two double-blind randomised cross-over trials

**DOI:** 10.1186/s40795-020-00365-4

**Published:** 2020-09-14

**Authors:** Yannis Manios, Eva Karaglani, Inge Thijs-Verhoeven, Elpis Vlachopapadopoulou, Anastasia Papazoglou, Eleni Maragoudaki, Zafeiris Manikas, Tarek-Michail Kampani, Iliana Christaki, Marlotte M. Vonk, Rolf Bos, Panam Parikh

**Affiliations:** 1 Department of Nutrition and Dietetics, School of Health Science and Education, Harokopio University, Athens, Greece; 2 FrieslandCampina, Stationsplein 1, 3818 LE Amersfoort, the Netherlands; 3 Department of Endocrinology-Growth and Development, Children’s Hospital P. & A. Kyriakou, Athens, Greece

**Keywords:** Milk fat, SN-2-palmitate, Palmitate soap, Calcium excretion, Stool consistency, Amsterdam infant stool scale

## Abstract

**Background:**

Palmitic acid (PA) is predominantly esterified at the SN-2 position of triacylglycerols in human milk. PA at the SN-2 position is more efficiently absorbed and results in reduced formation of PA soaps, as well as reduced fatty acid (FA) and calcium malabsorption. Bovine milk fat (MF), a natural source of SN-2-palmitate, was used in the fat blend of infant formulae (IF) in the current study to investigate its effect on stool fatty acid soaps, calcium excretion and stool characteristics.

**Methods:**

Two double-blind, randomised cross-over trials (CS1, CS2) were conducted in parallel with healthy term, formula-fed infants aged 9–14 weeks. After a two-week run-in period, infants in CS1 (*n* = 17) were randomly allocated to receive either a 50% MF-based formula (50MF) or a 100% vegetable fat (VF) formula; in CS2 (*n* = 18), infants received either a 20% MF-based formula (20MF) or the VF formula, in a 2 × 2-week cross-over design. At the end of each two-week intervention period, stool samples were collected for FA, FA soaps and calcium excretion analysis and stool consistency was assessed according to the Amsterdam Infant Stool Scale (AISS).

**Results:**

MF-based groups showed no significant difference in PA in stools compared to VF group, although reduced stool PA soaps (CS1: 111.28 ± 18.33 vs. 220.25 ± 29.35 mg/g dry weight, *p* < 0.0001; CS2: 216.24 ± 25.16 vs. 233.94 ± 35.12 mg/g dry weight, *p* = 0.0023), total FA soaps and calcium excretion (CS1: 46.40 ± 5.27 vs. 49.88 ± 4.77 mg/g dry weight, *p* = 0.0041; CS2: 46.20 ± 4.26 vs. 50.47 ± 6.71 mg/g dry weight, *p* = 0.0067) were observed. Furthermore, the 50MF group showed a favourable lower mean stool consistency score compared to the VF group (1.64 ± 0.49 vs. 2.03 ± 0.19, *p* = 0.0008).

**Conclusions:**

While the use of bovine MF in IF did not affect PA concentrations in stool, lower excretion of palmitate soaps, total FA soaps and calcium was seen in healthy term infants. 50MF formula also showed improved stool consistency. The use of MF in IF could be an interesting approach to improve gut comfort and stool characteristics in infants, warranting further research.

**Trial registration:**

Netherlands Trial Registry Identifier: NTR6702. Date registered: December 01, 2017.

## Background

Human milk (HM) represents optimum nutrition for full-term babies throughout infancy and is designed to meet the needs of the growing infant in the first months after birth [1]. Triacylglycerols (TAGs) in HM provide approximately 50% of the energy as well as essential fatty acids (FAs) important for the overall development of the infant [2–4]. Palmitic acid (PA), one of the major saturated fatty acids in HM (representing approximately 20–25% of total FAs), is predominantly esterified at the SN-2 position of TAGs (i.e. SN-2-palmitate) in HM. [1, 2, 5] Studies over the last two to three decades have provided increasing evidence that the SN-2-predominant positioning of PA in HM TAGs promotes the absorption of both PA and calcium in term and preterm infants [3, 6–8].

The majority of infant formulae (IF) use a blend of vegetable oils as a source of fat. Compared to HM fat, in which 70–88% of the PA is esterified at the SN-2 position, commonly used vegetable oils have lower percentage of PA in the SN-2 position of TAGs (10–20%) [5]. Therefore, vegetable fat (VF) blends consist of TAGs with PA predominantly bound to the SN-1 and SN-3 positions [5, 9]. During digestion, PA at the SN-1,3 positions is released as free PA. In the alkaline environment of the small intestinal lumen, free PA interacts readily with cations (e.g. calcium) to form insoluble soaps [10, 11] that are associated with hard stools, gut discomfort and decreased absorption of PA and minerals by the infant [8, 11, 12]. Increasing the ratio of SN-2 to SN-1 and SN-3 palmitate in IF could ensure higher absorption of fat and minerals (calcium), as well as lead to reduced formation of insoluble soaps, thereby, minimizing gut discomfort.

Synthetic structured TAGs have been developed with higher proportion of PA in the SN-2 position (ranging from 35.9–74%) and lower levels of PA at the SN-1 and SN-3 positions. Favourable effects of IF containing such synthetic TAGs on FA, calcium absorption and stool consistency have been reported in healthy infants by several studies [6, 7, 13–19].

Bovine milk fat (MF) is naturally higher in SN-2-palmitate than VFs, with a level of approximately 40% [8, 9, 11] and a higher ratio of SN-2 vs SN-1,3 palmitate. Furthermore, MF shows comparable TAG structures to those in HM fat [8]. Therefore, using MF in the development of IF may enable mimicking the composition and structure of HM fat, potentially leading to a higher absorption of PA and calcium, less soap formation and softer stools in comparison to IF containing VF only.

This paper reports on two studies. Each study was a double-blind, cross-over, randomised, placebo-controlled comparing a MF-based formula against a standard VF formula. The primary objective of these studies was to evaluate the excretion of PA and PA soaps in stools of healthy term infants. We hypothesised that infants fed MF-based IF had lower PA and PA soaps in stool when compared to infants fed VF-based formula. In addition, the secondary outcomes of both studies were calcium excretion in stools, stool consistency scores and other FA and FA soaps in stools.

## Methods

### Study design and population

**Fig. 1:**
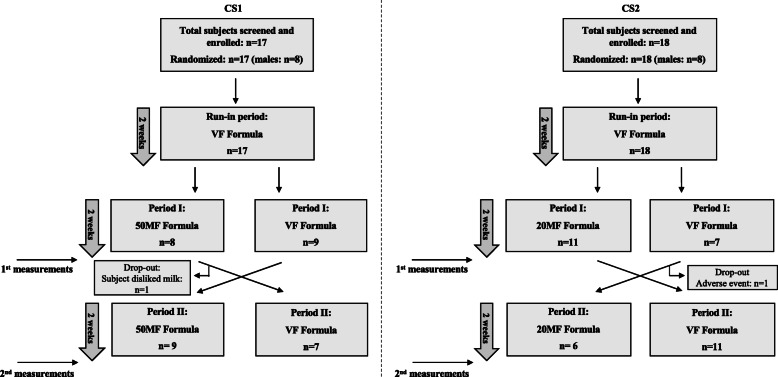
Study flowchart and subjects’ disposition CS1: cross-over study 1; CS2: cross-over study 2. MF: milk fat; VF: vegetable fat; 50MF: 50% MF formula; 20MF: 20% MF formula

The present studies were two separate double-blind, cross-over, randomised, placebo-controlled trials, conducted in parallel with healthy, full-term, exclusively formula-fed (FF) infants (Fig. 1). Sampling and recruitment were performed by paediatricians at 12 private paediatric clinics in two cities (Athens and Larissa) in Greece between December 2017 and July 2018. Infants were screened between their 9th–14th week of age on the following inclusion criteria: full-term, healthy (born at gestational age ≥ 37 weeks), exclusively FF infants, with appropriate for gestational age birthweight. Exclusion criteria were: i) severe acquired or congenital diseases, mental or physical disorders, any symptoms of allergy (including cow’s milk allergy); ii) Use of probiotics, antibiotics or other medication that treat or cause GI symptoms; iii) use of medication(s) known or suspected to affect fat digestion, absorption and/or metabolism, nutritional supplements, suppositories, medication that may suppress or neutralize gastric acid secretion and gut motility at the time of screening or at any time throughout the study period; iv) participation in another clinical trial; v) any type of mixed feeding (See eMethods 1 for full inclusion and exclusion criteria). Written informed consent was obtained from parents after explanation of the study procedures and prior to inclusion into the study. The study procedures were initiated immediately upon inclusion. The protocol, information letter to the parents/caregivers and written informed consent form were approved by Harokopio University’s Ethics Committee. The study was conducted in accordance with the guidelines of the Declaration of Helsinki and the International Conference on Harmonisation (ICH) guidelines on Good Clinical Practice (GCP) and was registered in the Netherlands Trial Registry (identifier: NTR6702).

### Study randomisation and formulae

Upon inclusion in the study, all infants were fed the 100% VF formula with 10.1% SN-2-palmitate levels (; total PA 24.9%) for 2 weeks (run-in period) in order to minimize the potential effects of previous feedings. Infants were then allocated to one of the cross-over studies using block randomisation. In each of the studies infants were randomly assigned to receive either the VF formula or a MF-based formula: i) 50% MF + 50% VF (50MF) with 39% SN-2-palmitate levels (total PA 18.9%) in cross-over study 1 (CS1) and ii) 20% MF + 80% VF (20MF) with 19.7% SN-2-palmitate levels (total PA 26.1%) in cross-over study 2 (CS2). Randomisation into the two treatment arms per study was based on a computer-generated sequence. After 2 weeks (period I), infants were crossed over to receive the other formula for another 2 weeks (period II) in their respective CS1 and CS2 (Fig. 1). The nutritional composition of the three study formulae was similar with the only difference being their FA profiles and percentage of SN-2-palmitate (Table 1). The procedures followed for the determination of SN-2-palmitate and total FA profile of study products can be found in eMethods 2. All powder properties were identical between the control and experimental formulae. All formulae were produced in the Netherlands by FrieslandCampina and were packaged in similar blank tins of 400 g each with a specific identification code at the bottom of the tins. The study formulae were labelled by the manufacturer using a single letter per formula group (A, B, C, D or E). The manufacturer retained the codes for the study formulae. All study personnel, including the Principal Investigator and the Sponsor’s Project Manager as well as parents/caregivers were blinded to the formulae allocation. Sealed envelopes containing product codes were provided to the study site in the event of an emergency. The tin label included guidance for the parents on the daily volume of formula intake required by the infant, which depended upon age and weight.

**Table 1:** Composition of the study formulae

	Formula		
Nutrient/ingredient	50MF	20MF	VF
Energy (kcal/100 mL)	66	66	66
Intact protein (g/100 mL)	1.4	1.4	1.4
Carbohydrates (g/100 mL)	7.1	7.0	7.0
Galacto-oligosaccharides (g/100 mL)	0.27	0.27	0.27
Fat (g/100 mL)	3.5	3.5	3.5
Docosahexanoic acid (mg/100 mL)	6.9	6.9	6.9
Arachidonic acid (mg/100 mL)	8.3	8.3	6.9
*Fatty acids; mol % of TAGs*			
C12:0; Lauric acid	6.0	7.7	10.4
C14:0; Myristic acid	7.4	4.8	3.9
C16:0; Palmitic acid	18.9	26.1	24.9
C18:0; Stearic acid	5.2	4.4	3.4
C18:1; Oleic acid	36.9	42.2	39.0
C18:2; Linoleic acid	11.7	16.4	12.7
C18:3; a-Linolenic acid	1.5	1.6	1.8
C20:0; Arachidic acid	0.2	0.3	0.3
% C16:0 in sn-2 position	39	19.7	10.1
Calcium (mg/100 mL)	53	55	56

*MF* milk fat; *VF* vegetable fat. *50MF* 50% MF formula; *20MF* 20% MF formula

To ensure double-blindness, all formulae were packaged in similar blank tins of 400 g each with different identification codes at the bottom of the tins. Formula labels provided preparation, storage and feeding instructions in English and Greek

### Stool collection and analysis

Stool samples were collected at home by parents/caregivers for three consecutive days at the end of period I and period II for analysis of their FAs, FA soaps and calcium content. Each freshly passed stool was placed in a faecal tube collector (until 30 g was collected in total), kept in a ziplock amber plastic bag and then stored in the home freezer. At the end of each intervention period, the study personnel collected the stool samples from the homes and brought them to Harokopio University. The stool samples were stored in Harokopio University in a freezer at -80 °C until being transported in dry ice to Covance Laboratory, Madison, Wisconsin, USA for analysis. The analytical procedures followed in the laboratory are described in eMethods 2.

### Formula consumption and stool characteristics

Parents/caregivers were asked to record formula consumption using a three-day milk diary, where the timing, frequency as well as the exact amount/volume (in mL) of formula consumed were recorded during the same 3 days of each intervention period as stool collection. Additionally, the study personnel collected all formula tins to monitor compliance and formula consumption.

Stool characteristics assessment was performed by parents/caregivers using the validated Amsterdam Infant Stool Scale (AISS) [20], which assesses the consistency, amount/volume and colour of stools. For assessment of consistency, each freshly passed stool during the three-day period was evaluated and ranked accordingly on a scale of one to four (watery = 1, soft = 2, formed = 3, hard =4) and a mean score was calculated.

### Safety and anthropometric assessment

Adverse events (AEs) and serious adverse events (SAEs) were recorded throughout the study and monitored by an independent paediatrician. No code-break requests occurred for AEs or SAEs throughout the study and de-blinding did not need to take place. Anthropometric indices (weight and length) were also measured following standardized procedures at screening and at the end of the run-in period, period I and period II.

### Statistical analysis

Sample size for both studies was determined based on the data from one available cross-over study by Carnielli et al. 1995 [14] on the concentration of PA in stools in infants fed control and high SN-2-palmitate formula, and adjusted for dose and duration. At least 16 infants per cross-over study were required to achieve a power of 80% (α = 0.05) to detect a mean (SD) between-group difference of 25 (13.9) mg PA per /g of wet stool between VF control IF and MF-based IF. Assuming an expected 30% drop-out rate, 22 infants per cross-over study were required to achieve 16 evaluable infants per cross-over study. Data analyses were performed with the study groups coded; the code was not broken until all analyses had been completed.

The two cross-over studies were analysed independently from each other by 4Pharma Ltd. (Finland) using SAS® version 9.4 for Windows (SAS Institute Inc., Cary, NC, USA). The primary outcomes were excretion of PA and PA soaps in stool. A hierarchical approach was taken when interpreting the results, with PA in stool tested first for statistical significance, followed by PA soaps in stool. Therefore, no further adjustments for multiplicity were conducted on the *p*-values. ANOVA appropriate for a 2 × 2 cross-over design was used to assess mean differences in stool PA and PA soap composition. When the normality assumption was not met, variables were log-transformed or Wilcoxon signed-rank test was applied. The statistical model included treatment, sequence and period as fixed effects, and subject (sequence) and residual error term as random effects.

The secondary outcomes were calcium absorption and stool consistency (using AISS). The same ANOVA approach was used for calcium excretion and stool consistency analysis. Milk intake comparisons between the formula groups was done using Mann-Whitney U-test. All statistical tests were two-sided and performed with α = 0.05.

Additional exploratory analyses were performed on total FA, total FA soaps, FA and FA soaps (ANOVA as with primary outcomes).

## Results

### Study population

From the total infants enrolled in CS1 and CS2 (*n* = 17 and *n* = 18, respectively), one infant dropped out of CS1 (subject disliked milk) and one from CS2 (subject had adverse event, not related to study product). The total number of infants that completed CS1 and CS2 was *n* = 16 and n = 17, respectively (Fig. 1). It was decided to stop recruitment when each cross-over study had at least 16 infants completing the study. The overall drop-out rate was below 10% (2 subjects dropped out).

The baseline and family characteristics of the subjects are descriptively presented in Table 2. Weight at birth, gestational age as well as infants age and weight at inclusion were similar among the groups per cross-over study.

**Table 2:** Baseline infant and family characteristics

	CS1		CS2	
	50MF - VF (*n* = 7)	VF – 50MF (*n* = 9)	20MF - VF (*n* = 11)	VF - 20MF (*n* = 6)
Gender, No. (%) male	3 (43)	5 (56)	6 (55)	2 (33)
Age at screening, mean (SD), days	103 (16)	92 (22)	95 (18)	96 (17)
Weight at screening, mean (SD), g	6368 (798)	5380 (1018)	5941 (1105)	5192 (722)
Mother’s age, mean (SD), years	34 (7)	32 (5)	35 (8)	33 (4)
Mother’s education level:				
No. (%) < 12 years	2 (29)	3 (33)	5 (46)	1 (17)
No. (%) 12–14 years	2 (29)	2 (22)	1 (9)	3 (50)
No. (%) > 14 years	3 (43)	4 (44)	5 (46)	2 (33)
Gestational age, mean (SD), weeks	39 (2)	38 (1)	39 (1)	38 (1)
Mode of delivery				
No. (%) caesarean section	4 (57)	7 (78)	6 (55)	5 (83)
Weight at birth, mean (SD), g	3259 (491)	2883 (391)	3143 (399)	2833 (318)

Data are descriptively summarized, given the cross-over design of the study

*CS1* cross-over study 1; *CS2* cross-over study 2; *SD* standard deviation; *50MF* 50% MF formula; *20MF* 20% MF formula; *MF* milk fat; *VF* vegetable fat

### Formula consumption and anthropometric data

The average weekly milk intake or the subjects’ weight and length measurements at the end of the two-week intervention periods did not differ between the MF and VF groups in either of the cross-over studies (eTable 3).

### Stool fatty acids

The faecal concentrations of the major FAs are reported in Table 3. No significant difference was noted in the PA in stool between the MF-based IF and VF formula in both, CS1 and CS2. Similarly, no difference was observed for the total free FAs between the MF-based IF and VF formula.

**Table 3:** Stool fatty acids, fatty acid soaps and calcium composition (mg/g stool dry weight)

CS1			CS2		
	50MF (*N* = 16)	VF (*N* = 16)		20MF (*N* = 17)	VF (*N* = 17)
Free Fatty Acids			Free Fatty Acids		
Palmitic acid (C16:0)^2^	4.4 (3.4–10.3)	5.7 (4.4–9.1)	Palmitic acid (C16:0)^3^	5.9 (3.8–13.4)	4.9 (3.8–7.3)
Lauric acid (C12:0)^2^	0.50 (0.28–0.78)^1^	1.38 (1.11–1.99)	Lauric acid (C12:0)^1^	1.30 (0.72)^2^	1.59 (0.840
Myristic acid (C14:0)^1^	1.35 (0.70)^2^	1.00 (0.59)	Myristic acid (C14:0)^3^	0.98 (0.66–1.59)^2^	0.79 (0.64–1.00)
Stearic acid (C18:0)^2^	1.83 (1.25–4.37)^2^	1.25 (0.93–1.84)	Stearic acid (C18:0)^3^	1.40 (0.92–2.94)^2^	0.99 (0.83–1.48)
Oleic acid (C18:1 n-9)^2^	4.80 (3.32–7.84)	5.01 (3.91–8.30)	Oleic acid (C18:1 n-9)^3^	6.65 (4.09–8.29)	5.70 (4.65–7.43)
Linoleic acid (C18:2)^2^	0.73 (0.46–1.36)	0.84 (0.45–1.46)	Linoleic acid (C18:2)^2^	0.93 (0.72–1.95)	0.88 (0.84–1.37)
Gamma Linolenic acid (C18:3 n-6)^1^	0.08 (0.02)^2^	0.07 (0.02)	Gamma Linolenic acid (C18:3 n-6)^1^	0.09 (0.04)	0.08 (0.02)
Alpha Linolenic acid (C18:3 n-3)^3^	0.07 (0.07–0.10)	0.07 (0.06–0.11)	Alpha Linolenic acid (C18:3 n-3)^2^	0.09 (0.07–0.19)	0.09 (0.08–0.15)
Arachidic acid (C20:0)^2^	0.10 (0.07–0.18)	0.10 (0.09–0.17)	Arachidic acid (C20:0)^3^	0.09 (0.07–0.17)	0.09 (0.08–0.12)
Total FAs^1^	22.37 (11.43)	23.16 (12.84)	Total FAs^3^	18.6 (15.7–32.7)	19.4 (15.3–22.3)
Fatty Acid Soaps			Fatty Acid Soaps		
Palmitic soap (C16:0)^1^	111.28 (18.33)^1^	220.25 (29.35)	Palmitic soap (C16:0)^1^	216.24 (25.16)^2^	233.94 (35.12)
Lauric soap (C12:0)^2^	1.76 (1.50–2.27)^1^	6.83 (5.74–7.67)	Lauric soap (C12:0)^1^	4.38 (1.27)^1^	7.34 (1.88)
Myristic soap (C14:0)^1^	10.82 (2.09)	11.24 (1.37)	Myristic soap (C14:0)^3^	11.90 (10.90–13.20)	12.20 (11.10–12.70)
Stearic soap (C18:0)^1^	50.92 (7.81)^1^	31.21 (4.78)	Stearic soap (C18:0)^3^	39.50 (38.40–46.40)^2^	36.40 (31.20–37.60)
Oleic soap (C18:1 n-9)^2^	10.02 (7.05–14.05)	8.72 (7.61–12.65)	Oleic soap (C18:1 n-9)^1^	10.10 (6.11)^2^	11.63 (7.29)
Linoleic soap (C18:2)^2^	1.11 (0.70–1.42)	1.13 (0.92–1.47)	Linoleic soap (C18:2)^1^	1.21 (0.70)^2^	1.57 (0.98)
Total FA soaps^1^	201.63 (34.79)^1^	290.19 (42.81)	Total FA soaps^1^	296.59 (31.29)^2^	311.18 (39.75)
Calcium			Calcium		
Stool calcium^1^	46.40 (5.27)^2^	49.88 (4.77)	Stool calcium^1^	46.20 (4.26)^2^	50.47 (6.71)

^1^Analysis of variance for variable in original scale of measurement. Data are presented as mean (SD)

^2^ Analysis of variance for log-transformed variable. Data are presented as median (IQR)

^3^Non-parametric analysis (Wilcoxon Signed Rank). Data are presented as median (IQR)

*P*-values indicated by a, *p* < 0.0001; b, *p* < 0.05 are not eligible for statistical significance according to pre-defined hierarchy

*CS1* cross-over study 1; *CS2* cross-over study 2; *50MF* 50% MF formula; *20MF* 20% MF formula; *MF* milk fat; *VF* vegetable fat; *SD* standard deviation; *IQR* inter-quartile range

The MF-based IF group in both cross-over studies had lower Lauric acid (C12:0) concentrations (CS1: *p* < 0.0001; CS2: *p* = 0.004) than VF group. In contrast, the opposite was observed for Myristic (C14:0) and Stearic (C18:0) in the MF-based IF groups (*p* < 0.05) in both, CS1 and CS2. The 50MF group (CS1) also had higher level of Gamma Linolenic acid than the VF group (p < 0.05).

In addition, Table 3 presents the faecal concentrations of the major FAs as the % of each FA within total free FAs lost in one g of dry stool. In CS1, the 50MF group had a decreased % of PA (*p* = 0.0003) and Lauric acid (*p* < 0.0001), and increased % of Myristic and Stearic acids (*p* < 0.0001) compared to the VF group. In CS2, no differences were observed in the % of PA, however, a decreased % of Lauric acid was observed in the 20MF group compared to the VF group (*p* = 0.0002).

### Stool fatty acid soaps

The MF-based IF groups in both CS1 and CS2 had a lower concentration of total FA soaps in stool than the VF group (Table 3; CS1: *p* < 0.0001; CS2: *p* = 0.0077). In CS1, the 50MF group had a lower concentration of PA soaps in stool compared to the VF group (*p* < 0.0001). Similar results were also noted in CS2, with lower PA soaps in the 20MF group (*p* = 0.0023). In CS1, Lauric acid (C12:0) soap concentrations were lower (*p* < 0.0001), whilst Stearic acid (C18:0) soap concentration was increased in the 50MF group compared to the VF group (*p* < 0.0001). In CS2, a decrease in Lauric (C12:0), Oleic (C18:1) and Linoleic acid (C18:2) soap concentrations were observed in the 20MF group compared to the VF group (*p* < 0.05). Stearic acid (C18:0) soap concentration, however, was increased (*p* = 0.0021) (Table 3).

In addition, Table 4 presents the faecal concentrations of the major FA soaps as the % of each FA soap within total FA soaps lost in one g of dry stool. In CS1 and CS2 both, 50MF and 20MF groups had decreased % of PA soaps compared to the VF group (CS1: *p* < 0.0001; CS2: *p* = 0.0032). In CS1, similar results were observed for the % of Lauric acid (C12:0) soaps (*p* < 0.0001), while the opposite was observed for Myristic (C14:0), Stearic (C18:0) and Oleic acid (C18:1) soaps (*p* < 0.0001). In CS2, a decrease was observed for the % of Lauric (C12:0) and Linoleic acid (C18:2) soaps (*p* < 0.0001 and *p* = 0.0059, respectively), while the opposite was observed for Myristic (C14:0) and Stearic acid (C18:0) soaps (*p* = 0.0058 and *p* = 0.0026, respectively).

**Table 4:** Percentages of individual FAs and FA soaps within total free FAs and total FA soaps, respectively

CS1			CS2		
	50MF (*N* = 16)	VF (*N* = 16)		20MF (*N* = 17)	VF (*N* = 17)
% Individual Fatty Acids within Total Free FAs			% Individual Fatty Acids within Total Free FAs		
% Palmitic acid (C16:0)^1^	28.79 (8.41)^2^	35.88 (10.46)	% Palmitic acid (C16:0)^3^	31.2 (23.0–36.0)	29.3 (24.3–36.0)
% Lauric acid (C12:0)^1^	2.39 (0.73)^1^	7.05 (1.94)	% Lauric acid (C12:0)^1^	4.99 (1.78)^2^	7.28 (2.25)
% Myristic acid (C14:0)^1^	6.06 (1.01)^1^	4.26 (0.56)	% Myristic acid (C14:0)^1^	4.44 (0.92)	4.08 (0.62)
% Stearic acid (C18:0)^1^	11.57 (3.96)^1^	7.20 (1.94)	% Stearic acid (C18:0)^3^	7.43 (5.64–8.23)	5.73 (5.45–6.76)
% Oleic acid (C18:1 n-9)^1^	29.74 (10.25)	28.23 (9.07)	% Oleic acid (C18:1 n-9)^1^	31.11 (7.95)	28.51 (7.71)
% Linoleic acid (C18:2)^1^	4.66 (2.63)	4.39 (1.84)	% Linoleic acid (C18:2)^1^	5.68 (2.47)	5.79 (2.54)
% Gamma Linolenic acid (C18:3 n-6)^2^	0.37 (0.30–0.57)	0.35 (0.27–0.41)	% Gamma Linolenic acid (C18:3 n-6)^2^	0.32 (0.26–0.47)	0.35 (0.32–0.47)
% Alpha Linolenic acid (C18:3 n-3)^1^	0.50 (0.215)	0.44 (0.18)	% Alpha Linolenic acid (C18:3 n-3)^1^	0.52 (0.23)	0.57 (0.27)
% Arachidic acid (C20:0)^1^	0.58 (0.15)	0.59 (0.19)	% Arachidic acid (C20:0)^2^	0.52 (0.46–0.60)	0.54 (0.46–0.64)
% Fatty Acid Soaps within Total FA Soaps			% Fatty Acid Soaps within Total FA Soaps		
% Palmitic soap (C16:0)^3^	54.4 (54.1–57.3)^1^	76.2 (75.6–77.6)	% Palmitic soap (C16:0)^3^	72.7 (71.5–74.7)^2^	76.6 (74.0–77.3)
% Lauric soap (C12:0)^1^	0.93 (0.25)^1^	2.46 (0.38)	% Lauric soap (C12:0)^1^	1.48 (0.42)^1^	2.36 (0.53)
% Myristic soap (C14:0)^1^	5.36 (0.35)^1^	3.89 (0.15)	% Myristic soap (C14:0)^3^	4.05 (3.88–4.12)^2^	3.93 (3.71–3.96)
% Stearic soap (C18:0)^2^	25.52 (23.95–26.48)^1^	10.73 (10.34–11.01)	% Stearic soap (C18:0)^3^	14.04 (13.10–14.87)^2^	11.24 (10.31–11.78)
% Oleic soap (C18:1 n-9)^1^	5.45 (2.17)^2^	3.94 (1.88)	% Oleic soap (C18:1 n-9)^1^	3.38 (1.77)	3.72 (2.14)
% Linoleic soap (C18:2)^1^	0.62 (0.29)	0.50 (0.26)	% Linoleic soap (C18:2)^1^	0.40 (0.21)^2^	0.51 (0.29)

^1^ Analysis of variance for variable in original scale of measurement. Data are presented as mean (SD)

^2^ Analysis of variance for log-transformed variable. Data are presented as median (IQR)

^3^ Non-parametric analysis (Wilcoxon Signed Rank). Data are presented as median (IQR)

*P*-values indicated by a, *p* < 0.0001; b, *p* < 0.05 are not eligible for statistical significance according to pre-defined hierarchy.

*CS1* cross-over study 1; *CS2* cross-over study 2; *50MF* 50% MF formula; *20MF* 20% MF formula; *MF* milk fat; *VF* vegetable fat; *SD* standard deviation; *IQR* inter-quartile range

### Stool calcium

The mean calcium concentration in stools was lower in both 50MF and 20MF groups compared to their respective VF group (CS1: *p* = 0.0041; CS2: *p* = 0.0067; Table 3).

### Stool consistency

**Fig. 2:**
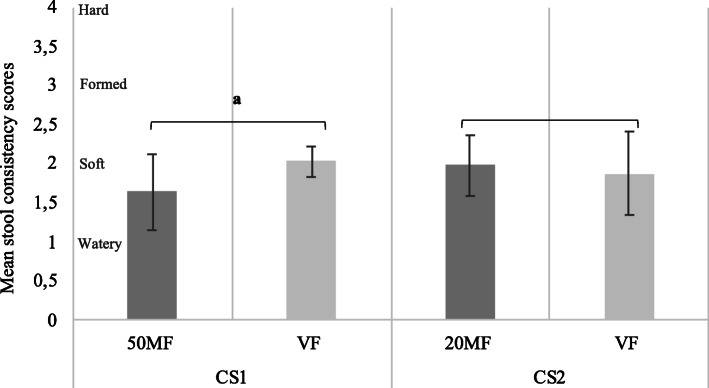
Stool consistency scores according to feeding group Individual stool consistency scores were determined using the Amsterdam Infant Stool Scale (AISS) (categorization: 1 = watery, 2 = soft, 3 = formed, and 4 = hard). Comparisons between the formula groups were conducted using analysis of variance. Values are mean (SD) CS1: cross-over study 1; CS2: cross-over study 2. MF: milk fat; VF: vegetable fat; SD standard deviation. Significant difference between the 50MF and the VF group: ^a^*p* = 0.0032; 50MF: 50% MF formula; 20MF: 20% MF formula

The mean stool consistency is presented in Fig. 2. In CS1, the mean stool consistency score was decreased in 50MF group compared to the VF group (*p* = 0.0032). Parents/caregivers of infants in the 50MF group reported watery and soft stools, while the VF group reported only soft stools. The mean stool consistency score in CS2 did not differ between the 20MF and VF groups, and was classified as soft.

## Discussion

To our knowledge, this is the first study assessing the effect of IF with bovine MF on stool FAs, FA soaps and calcium excretion in healthy term infants. Although, current studies did not show a significant difference on PA in stool as initial primary outcome measure, an interesting observation is that both, 50MF and 20MF formulae did demonstrate favourable effects on PA soaps in stool and other secondary outcomes, e.g. calcium excretion and total FA soaps in stools, compared to the VF formula. This underlines the importance of further exploration of bovine MF application in IF. Additionally, various FA showed different trends in FA soap concentrations with increase of MF content in the IF. As the IF in the current study differed in their overall FA profile, it is likely that this contributed to the observed FA trends and not just their distribution over SN-2 and SN-1,3 positions.

Interestingly, 50MF formula with high SN-2-palmitate levels favourably affected infants’ stool consistency scores. These findings are in line with published literature, although the reported studies had different study designs, age groups of infants and/or duration of interventions [6, 7, 14–16, 18, 19]. Most of these studies have tested IF with synthetic TAGs at various proportions of SN-2-palmitate, in contrast to the current MF-based formulae.

All previous studies consistently report that a higher SN-2-palmitate content in IF results in improved PA and FAs absorption [14, 15, 18] or lower faecal excretion, either as free PA and free FAs [6, 14] or as PA soaps and FA soaps in the faeces [6, 7, 16, 19]. No differences were observed between the current test groups and their respective control group on the absolute PA concentrations in the faeces, only the proportion of PA within total FAs excreted in the faeces was lower in the 50MF group compared to the VF group. However, infants fed with both MF-based formulae, despite lower SN-2-palmitate levels than reported in literature for synthetic TAGs [6, 7, 14–16, 18, 19], had lower amounts of PA soaps in their stools compared to the VF formula. Furthermore, infants fed 20MF also had lower faecal excretion of Oleic and Linoleic soaps compared to those receiving VF formula which can be speculated as an additional benefit of the increased SN-2-palmitate content using MF on the absorption of these essential FAs. This suggests that increasing the SN-2-palmitate content through the use of MF might have comparable favourable effects to synthetic TAGs even at a lower concentration.

Calcium excreted in the faeces was found to be lower in both MF groups compared to the VF group. This potentially suggests improved calcium absorption by the infants as reported by previous balance studies [14, 15, 18]. This finding is particularly relevant since the groups had comparable average IF intake and the calcium content in the formulae was similar. The potential health benefits of improved calcium availability on bone indices have been reported by two previous studies in healthy term infants which showed improved bone mass / bone strength / quality (as determined either by dual-energy x-ray absorptiometry [16] or by quantitative ultrasound measurements of bone speed of sound [21]) when a high (50 and 43%, respectively) SN-2-palmitate formula was used compared to a standard low (12 and 14%, respectively) SN-2-palmitate formula. A balance study to confirm whether the reduced faecal calcium excretion seen in this study correlates with improved calcium retention and absorption is warranted.

In this study we have used the AISS [20], which is considered a more appropriate tool for infants defecating in nappies [22] to assess stool consistency in SN-2-palmitate IF related studies. In general, FF infants have harder stools compared to breast-fed (BF) infants who typically have watery to soft stools [12]. Differences in stool consistency have been mainly associated with the higher content of FA soaps in the faeces of FF infants compared to the BF ones [12]. Results from previous studies, using different stool scales to assess the effect of IF with various SN-2-palmitate content on stool consistency, have been inconsistent. Two studies found that infants receiving a high (50 and 36%, respectively) SN-2-palmitate formula had softer, less-formed stools than infants in the low (12 and 12%, respectively) SN-2-palmitate formula groups [16, 19]. In contrast, the study by Nowacki et al. 2014 [7] showed no differences between the high (39%) and the low (13%) SN-2-palmitate groups. The study by Carnielli et al. 1996 [15] showed that infants fed the high (66%) SN-2-palmitate formula had a more favourable stool consistency score than the intermediate (39%) and low (13%) SN-2-palmitate formulae. Infants fed the intermediate formula had stool consistency scores between those of the high and the low SN-2-palmitate formulae [15]. In the present study, infants consuming the 50MF formula had a mean score closer to the watery category (which is similar to the BF infants [12, 23]) and the infants consuming the VF formula had a mean score closer to the soft category, while no differences were observed for the 20MF formula vs. the VF group. The lack of difference between the 20MF formula and VF formula could be explained by the absence of hard stool reports in any of the treatment groups, which might have limited the treatment effect induced by the 19.7% SN-2-palmitate levels in 20MF formula on stool consistency. Future studies including a reference group of BF infants may provide useful and relevant insights into stool consistency of infants.

## Conclusions

In summary, while the MF-based IF did not affect the concentrations of PA in stool, our studies demonstrate that increasing SN-2-palmitate in IF using bovine MF results in lower palmitate soaps, total fatty acid soaps and calcium excretion in stools in healthy, term infants. Furthermore, a favourable effect on stool consistency is also noticed with the 50MF IF. The present studies suggest a role for application of bovine MF in IF. Further research to validate these favourable effects, taking into account stereospecificity of the triglyceride, and with the inclusion of a BF reference group is warranted.

## Supplementary information

**Additional file 1.** Inclusion and exclusion criteria.

**Additional file 2.** Biochemistry analysis.

**Additional file 3.** Formula consumption and anthropometric data at the end of the two-week intervention periods.

## Data Availability

The datasets used and/or analysed during the current study are available from the corresponding author on reasonable request.

## Abbreviations

AISS: Amsterdam Infant Stool Scale
BF: Breastfed
CS1: Cross-over study 1
CS2: Cross-over study 2
FA: Fatty acid
FF: Formula-fed
HM: Human milk
IF: Infant formula
MF: Milk fat
PA: Palmitic acid
TAG: Triacylglycerol
VF: Vegetable fat
