# Supplementary material for: Effect of milk fat-based infant formulae on stool fatty acid soaps and calcium excretion in healthy term infants: two double-blind randomised cross-over trials
Source: BMC Nutr. 2020 Sep 14;6:46. doi: 10.1186/s40795-020-00365-4 (PMC7489008; doi:10.1186/s40795-020-00365-4)
Supplement: Supplementary file 1 — Additional file 1. Inclusion and exclusion criteria. [file 40795_2020_365_MOESM1_ESM.docx]

**Additional file 1.** **Inclusion and exclusion criteria**

| **Inclusion criteria:**   - Full-term, healthy infants (born at gestational age ≥37 weeks). - “Appropriate for gestational age” birthweight (i.e. 10^th^ centile ≤ Birth weight ≤ 90^th^ centile). - Age at enrolment: between 9^th^-14^th^ week. - Exclusively formula fed infants before and during the entire intervention period. - Parents willing and agreeing to initiate complementary feeding after the end of endpoint measurements, i.e. after the completion of the 5^th^ month of age. - Parents willing to collect stools and fill in all study questionnaires and diaries during the entire intervention period. - Written informed consent.   **Exclusion criteria:**   - Severe acquired or congenital diseases, mental or physical disorders, any symptoms of allergy (including cow’s milk allergy). - Parents or siblings with documented CMA allergy, diagnosed by a doctor. - Use of probiotics, antibiotics or other medication that treat or cause GI symptoms and/or affect appetite at the time of screening or at any time throughout the study period (these infants will be considered as drop-outs). - Use of medication(s) known or suspected to affect fat digestion, absorption and/or metabolism; nutritional supplements; suppositories; medication that may suppress or neutralize gastric acid secretion and gut mobility at the time of screening or at any time throughout the study period (these infants will be considered as drop-outs). - Participation in another clinical trial. - Any type of mixed feeding (i.e. combination of formula with breastfeeding in any proportion) and/or complementary feeding during the intervention. |
| --- |
