# Supplementary material for: Effect of milk fat-based infant formulae on stool fatty acid soaps and calcium excretion in healthy term infants: two double-blind randomised cross-over trials
Source: BMC Nutr. 2020 Sep 14;6:46. doi: 10.1186/s40795-020-00365-4 (PMC7489008; doi:10.1186/s40795-020-00365-4)
Supplement: Supplementary file 2 — Additional file 2. Biochemistry analysis. [file 40795_2020_365_MOESM2_ESM.docx]

**Additional file 2. Biochemistry analysis**

| Prior to analysis of the stool samples, the samples were thawed, pooled, homogenized and lyophilized, and the % moisture was determined gravimetrically. The dried samples were extracted by solvent reflux to obtain the neutral lipids, including non-soaped free FA. The remaining samples were treated with acetic acid to release the soaped FA which were isolated by a second solvent reflux step. The free acids were isolated using solid phase extraction. The free acids were then converted to methyl esters using methanolic hydrochloric acid. The resulting FA methyl esters were analyzed using a gas chromatograph equipped with a flame ionization detector and quantitated using external standards. Total FA soaps were calculated from the sum of all measured individual FA soaps. Both free FAs and soaped FAs were reported as mg/g dry weight stool in the acid form. Stool calcium content was determined by Inductively Coupled Plasma Emission Spectrometry using the AOAC International Official Methods of Analyses protocol^1^.  Determination of SN-2-palmitate and total FA profile of Study products. The fat structure in IFT formulas was determined according to a SN-1/3 specific pancreatic lipase-based hydrolysis of TAG^2^. The 2-monoacylglycerols formed are isolated by thin layer chromatography and are subsequently methylated for gas chromatographic analysis (GC) and quantified in weight concentrations FA methyl esters (FAME). The latter is done by standard ISO methods^3,4^. For the conversion to molar FA concentrations, corrections are made for the FAME molecular weights.  **References**  ^1^Pacquette LH, Thompson JJ, Malaviole I, et al. Minerals and Trace Elements in Milk, Milk Products, Infant Formula, and Adult/Paediatric Nutritional Formula, ICP-MS Method: Collaborative Study, AOAC Final Action 2015.06, ISO/DIS 21424, IDF 243. *J AOAC Int*. 2018;101(2):536-561. |
| --- |

^2^Luddy, F. E., Barford, R. A., Herb, S. F., Magidman, P., & Riemenschneider, R. W. (1964). Pancreatic lipase hydrolysis of triglycerides by a semimicro technique. Journal of the American Oil Chemists Society, 41(10), 693–696. <https://doi.org/10.1007/BF02661412>

^3^ISO15884 / IDF182. (2002). Milk fat – Preparation of fatty acid methyl esters.

^4^ISO15885 / IDF184. (2002). Milk fat – Determination of the fatty acid composition by gas-liquid chromatography.
