# Supplementary material for: Effect of milk fat-based infant formulae on stool fatty acid soaps and calcium excretion in healthy term infants: two double-blind randomised cross-over trials
Source: BMC Nutr. 2020 Sep 14;6:46. doi: 10.1186/s40795-020-00365-4 (PMC7489008; doi:10.1186/s40795-020-00365-4)
Supplement: Supplementary file 3 — Additional file 3. Formula consumption and anthropometric data at the end of the two-week intervention periods. [file 40795_2020_365_MOESM3_ESM.docx]

**Additional file 3. Formula consumption and anthropometric data at the end of the 2-week intervention periods**

|  | **CS1** | |  | **CS2** | |  |
| --- | --- | --- | --- | --- | --- | --- |
|  | **50MF (N=16)** | **VF (N=16)** | **p-value** | **20 MF**  **(N=17)** | **VF (N=18)** | **p-value** |
| Average weekly milk intake, mean (SD), mL | 5707 (814) | 6063 (1009) | 0.28 | 5763 (1300) | 6232 (1230) | 0.3 |
| Weight, mean (SD), g | 6807.13 (918.45) | 6706.25 (1089.85) | 0.87 | 6566.35 (1047.99) | 6697.78 (1139.31) | 0.78 |
| Length, mean (SD), cm | 64.48 (2.74) | 64.28 (3.71) | 0.93 | 64.28 (2.87) | 65.14 (3.18) | 0.64 |
| Comparisons between the formula groups were conducted using Mann-Whitney U-test.  CS1: cross-over study 1; CS2: cross-over study 2; 50MF: 50% MF formula; 20MF: 20% MF formula; MF: milk fat; VF: vegetable fat; SD: standard deviation. | | | | | | |
